# Betaine and Choline Improve Lipid Homeostasis in Obesity by Participation in Mitochondrial Oxidative Demethylation

**DOI:** 10.3389/fnut.2018.00061

**Published:** 2018-07-10

**Authors:** Sugashan Sivanesan, Adrian Taylor, Junzeng Zhang, Marica Bakovic

**Affiliations:** ^1^Department of Human Health and Nutritional Sciences, University of Guelph, Guelph, ON, Canada; ^2^Aquatic and Crop Resource Development, National Research Council Canada, Halifax, NS, Canada

**Keywords:** betaine, choline, methyl donors, mouse models, obesity

## Abstract

We investigated the metabolic effects of betaine (Bet) supplementation on CTP:phosphoethanolamine cytidylyltransferase/Pcyt2 heterozygous mice (HET). HET received either no treatment or were allowed access to 1% Bet supplemented water for 8 weeks. As we previously showed with choline (Cho), Bet improved hypertriglyceridemia, and hepatic steatosis in HET. The protection from obesity associated with reduced hepatic steatosis and increased lipid breakdown in adipocytes was attributed to increased energy requirements for metabolism and elimination of supplemented Bet and Cho. ^1^H-NMR-based profiling revealed metabolic changes caused by Bet and Cho supplementation. Cho increased the citric acid cycle intermediate succinic acid while reducing isoleucine, valine, threonine, and lysine. Bet increased α-ketoglutaric acid and did not stimulate catabolism of amino acids. Increased histidine and alanine are specific biomarkers for Bet treatment. Cho and Bet caused glycerol accumulation and reduced sarcosine, taurine, acetate, and β-hydroxybutyrate levels. These data provide new insights on how Cho and Bet supplementation can aid in treatment of obesity related disorders due to their positive effects on lipolysis, the citric acid cycle, and mitochondrial oxidative demethylation.

## Introduction

Choline (Cho) is an essential nutrient required for fetal development, neuronal function, as well as protection against liver and muscle cell death (1, 2). Cho has multiple roles in metabolism including incorporation into membrane phospholipids such as phosphatidylcholine (PC) and sphingomyelin, neurotransmitter formation (i.e., acetylcholine), and mitochondrial oxidation to betaine (Bet), a critical osmolyte and methyl-group donor in one carbon metabolism. Most dietary Cho is converted to PC, is released from PC by lipolysis, and recycled in distant tissues. Cho is irreversibly metabolized to Bet by mitochondrial oxidation and this process may be downregulated when Cho supply is low or when demands for Cho are high such as during development, pregnancy, and lactation. Additional PC is obtained endogenously by the methylation of phosphatidylethanolamine (PE) by S-adenosylmethionine (SAM) and PE methyltransferase (PEMT) (3). This pathway is active predominantly in the liver and is important for the formation and secretion of bile and lipoprotein PC but cannot completely fulfill the requirements of dietary Cho (4).

Genetic polymorphisms and epigenetic influences on dietary requirements of Cho and Bet are increasingly a focus of human research. Additionally, biomarkers of Cho and Bet status to predict the risk for chronic diseases and provide a basis for human intervention trials are urgently needed (4, 5). It is established that genetic variants of choline metabolizing enzymes CHKA, CHDH, PEMT, and choline transporter SLC44A1 determine the use of dietary Cho as a methyl group donor, partitioning of dietary Cho between Bet and PC synthesis, and distribution of dietary Cho between the two separate pathways for PC synthesis; the PEMT pathway and the CDP-choline pathway (5). Thus, the establishment of biomarkers for dietary intake of Cho and Bet is increasingly relevant for diseases affecting Cho and Bet metabolism such as non-alcoholic fatty liver disease (NAFLD), diabetes and atherosclerosis. This is especially relevant in the context of individual differences that may contribute to disease pathogenesis over the long-term.

Current understanding of the mechanisms and metabolic changes during Cho and Bet supplementation are still incomplete. A higher intake of Cho improves body composition (6) and cognitive performance (7), and in postmenopausal women with NAFLD decreased Cho intake is associated with increased liver fibrosis (8). Cho and Bet consumption are inversely correlated with glucose and insulin sensitivity and occurrence of metabolic disorders (9). In contrast to Cho, Bet has been increasingly recognized as an effective therapy for alcoholic and non-alcoholic steatohepatitis (NASH) (10, 11).

Metabolism of Cho and Bet occurs in mitochondria by oxidative demethylation pathway that requires oxygen and reducing equivalents from the TCA cycle/electron transport chain Therefore, we anticipate that supplementation of Cho and Bet will raise metabolic demands for their degradation and consequently reduce obesity. We characterize the effects of dietary Cho and Bet in the CTP:phosphoethanolamine cytidylyltransferase/Pcyt2 heterozygous (HET) mouse model developed in our laboratory (12–16). In our previous work we showed that plasma free fatty acids and cholesterol are not modified but that triglycerides (TAG), glucose, and insulin are elevated in HET mice (12). This mouse is unable to efficiently synthesize PE from ethanolamine and diacylglycerol, which causes a homeostatic shift toward increased fatty acid/TAG synthesis and inhibited lipolysis/ fatty acid (FA) oxidation, resulting in NAFLD, insulin resistance, hypertriglyceridemia, and obesity (12). Cho administration to HET mice stimulates lipolysis and FA oxidation via PPARα and PGC-1α activation, thereby preventing disease development and weight gain (13, 14). Cho increases PC remodeling but does not modify the PC and PE content or expression of liver and adipocyte phospholipid genes (13). Similarly, the positive effect of Cho on muscle FA/TAG metabolism is accompanied by the activation of AMPK and PI3K/Akt insulin signaling pathway and improvements in glucose stores in HET muscle (14).

Here, we hypothesize that if the oxidative demethylation pathway is responsible for the lipid lowering effects of Cho, Bet supplementation will then reduce NAFLD, and consequently obesity with a similar mechanism. We characterized the plasma metabolic profiles of HET mice over the course of Bet and Cho treatments using ^1^H-NMR-based metabolomics and identify common and specific metabolites of Cho and Bet supplementation. Our goal was to establish the systemic effects of Cho and Bet supplementation and to identify the circulating biomarkers for their treatments that could be translated in future dietary intervention studies.

## Results

### Bet supplementation alleviates liver steatosis, obesity and hypertriglyceridemia

**Figure 1:**
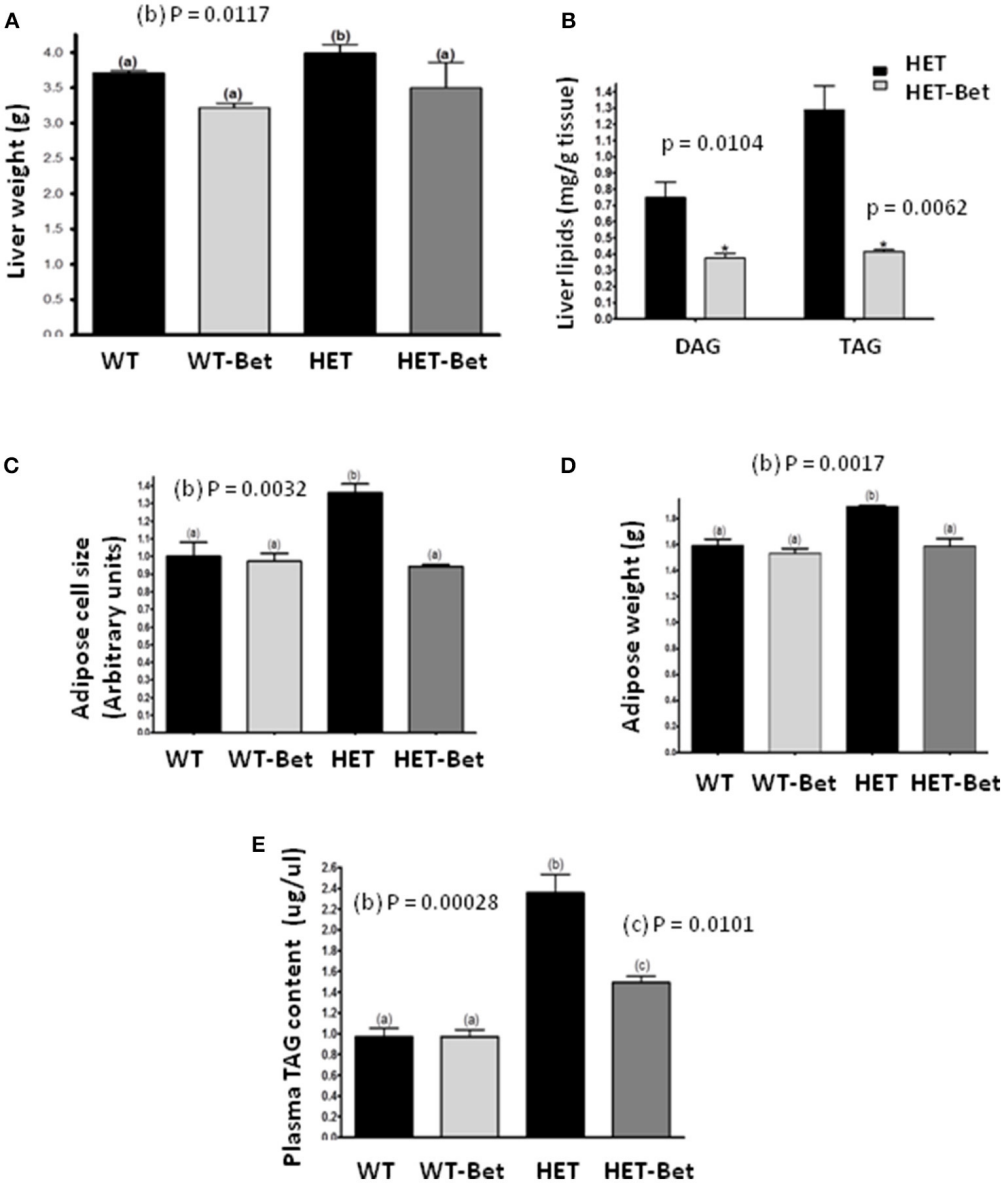
Betaine supplementation decreases liver and adipose tissue weight and lipid content. **(A)** Liver weight (*n* = 6 per group) was decreased in Bet treated HET mice (HET-Bet) but not in the wild-type treated (WT-Bet) mice. **(B)** Bet reduced HET liver (*n* = 8) DAG and TAG by 2- and 3-fold, respectively. Betaine decreased adipose cell size **(C)** and tissue weight **(D)** to the levels observed in WT and WT-Bet mice (*n* = 5 per group); **(E)** HET mice plasma TAG compared to lean WT mice was ~2-fold higher. Plasma lipid content (*n* = 6) was decreased by 40% in HET-Bet and it was not modified in WT-Bet mice. Data analysis was performed by two-way ANOVA **(A, C–E)** and Student's *t*-test **(B)**. The groups sharing the same letter (*a*) are not statistically different from each other and the groups with different letters (*b* or *c*) are statistically different at indicated *p*-values.

**Figure 2:**
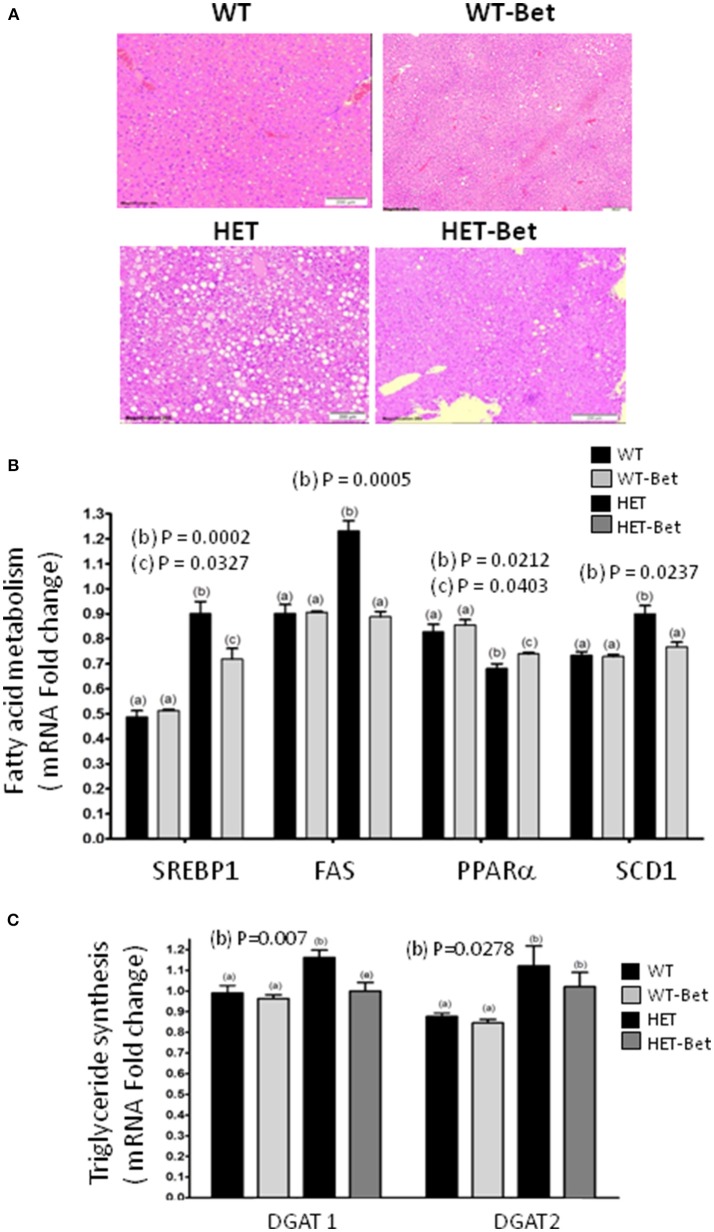
Betaine reduces liver steatosis and alters hepatic gene expression. **(A)** The fatty liver (*n* = 4) was considerably reduced (HET vs. HET-Bet) after 8 weeks of 1% betaine supplementation. WT livers (*n* = 8) did not have accumulation of lipid droplets and no change was observed after Bet treatment (WT-Bet). Histological analysis was performed on the livers fixed in paraffin and then stained with hematoxylin and eosin. **(B)** The effect of Bet on the expression of hepatic **(B)** sterol regulatory element-binding protein (Srebp1c), fatty acid synthase (Fas), stearoyl-CoA desaturase 1 (Scd1) and peroxisome proliferator-activated receptor alpha (Pparα) genes and **(C)** diacylglycerol acyltransferase 1 and 2 (Dgat1 and Dgat2) genes. Liver RNA (*n* = 3 per group) was isolated from Bet supplemented and control groups and gene expression determined relative to glycerol-3-phosphate dehydrogenase (Gapdh) as an endogenous control. Groups sharing the same letter are not statistically different from each other and the groups with different letters are statistically different from each other (two-way ANOVA; with *p*-values indicated).

Pcyt2 heterozygous mice (HET) progressively gain weight and develop hypertriglyceridemia, liver steatosis, and obesity (12–16). In order to elucidate the effects of Bet on obesity, mice were separated into untreated groups (WT and HET), and treated groups (WT-Bet and HET-Bet) that were allowed access to 1% Bet supplemented water for 8 weeks. After 8 weeks of treatment, the effects of Bet on liver DAG, and TAG levels, adipocyte weight/cell size, and plasma lipid content were established (Figures 1, 2).

As shown previously (12), the livers of HET mice were significantly heavier than WT littermates (*P* = 0.01; Figure 1A). Bet reduced liver weight in the HET-Bet group relative to the untreated HET group to become similar to WT and WT-Bet liver weights. With respect to weight reduction with Bet treatment, liver DAG (*p* = 0.01), and TAG (*p* = 0.006) were decreased by 2-fold and 3-fold respectively (Figure 1B). Adipocyte size and adipose weight of HET-Bet mice were decreased by 30% (*p* = 0.003) and 20% (*p* = 0.02) respectively and reached the WT levels (Figures 1C,D). HET mice plasma TAG compared to lean WT mice was ~2-fold higher (*p* = 0.0003). Plasma TAG levels did not change after Bet supplementation in WT mice however, Bet lowered the elevated TAG in HET plasma by 40% (*p* = 0.01) (Figure 1E). Histological analysis of Bet treated and control livers is shown in Figure 2A. In accordance with the increased liver size and elevated liver lipids (Figures 1A,B), histological analysis revealed large vacuole-like particles that correspond to lipid droplets, demonstrating severe liver steatosis in untreated HET mice (Figure 2A). Bet supplementation (HET-Bet) reduced liver steatosis and no visual differences in liver histology were observed between WT and WT-Bet mice.

### Bet normalizes expression of FA metabolic genes

**Figure 3:**
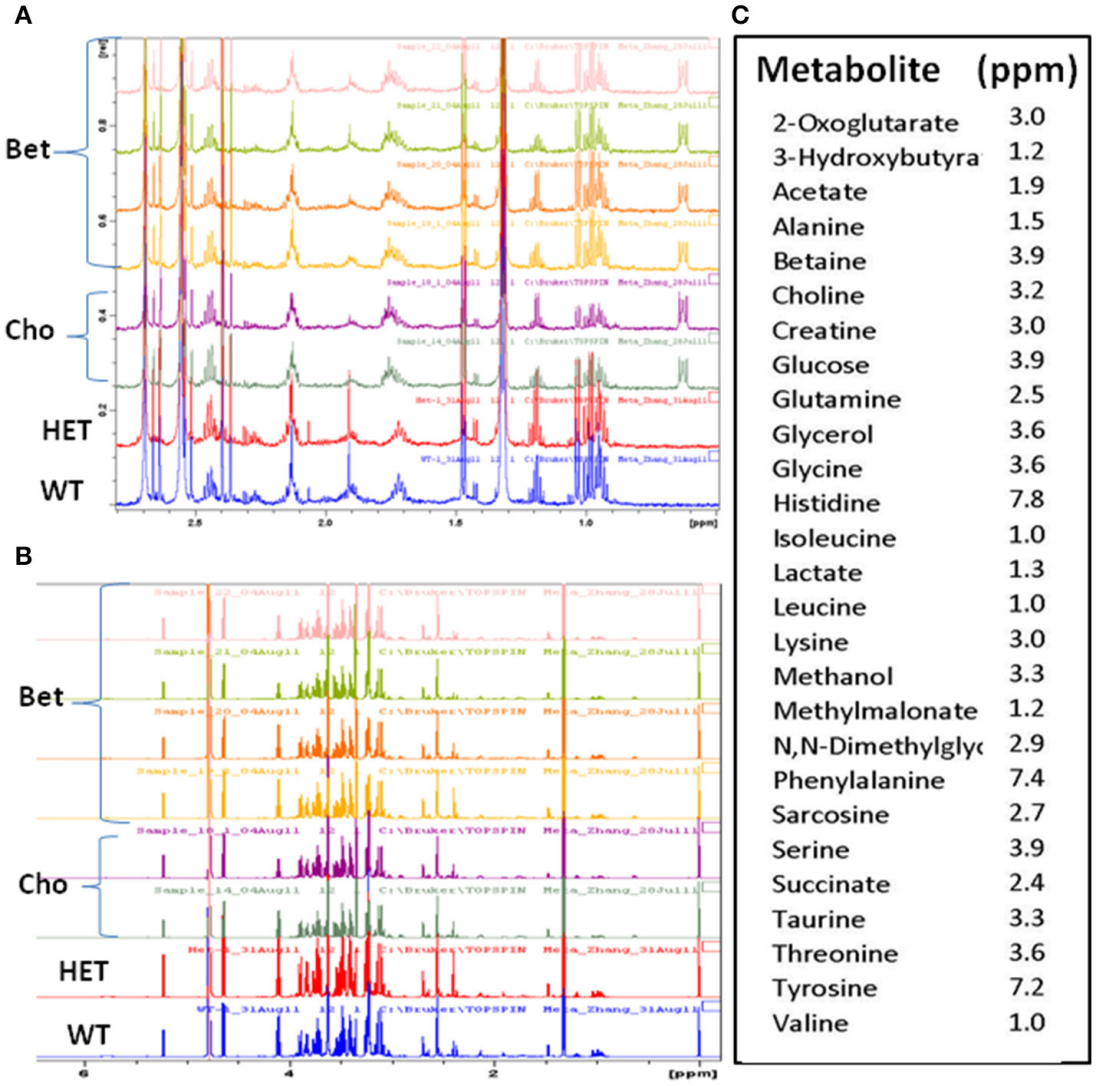
The overlaid ^1^H-NMR spectra of plasma samples for controls and supplemented groups. Comparisons of the representative spectra of the lean (WT), obese (HET), and betaine (Bet) and choline (Cho) supplemented plasma samples for **(A)** 0.5–2.8 ppm and **(B)** 1–6 ppm ^1^H-NMR regions; **(C)** the signature peaks (ppm) of the identified metabolites used in the targeted metabolite profiling.

HET mice are hyperglycemic and have elevated synthesis of FA from glucose by the process of lipogenesis as well as impaired FA oxidation in the mitochondria (12). To establish the effect of Bet on liver lipid metabolism, the expression of the main lipid genes was compared (Figures 2B,C). The expression of Srebp1c, Fas and Scd1 was increased 40% (*p* = 0.002), 30% (*p* = 0.0005), and 20% (*p* = 0.024) respectively in HET liver. As seen in Cho treatments (13), Bet reduced Srebp1c expression (*p* = 0.034) and normalized the expression of Fas and Scd1 genes in HET-Bet liver to the levels in the WT and WT-Bet livers. On the other hand, as shown previously (15) PPARα was significantly reduced in the liver of HET (*p* = 0.02, Figure 2B). Similarly to Cho treatments (13), Bet modestly increased (*p* = 0.04, Figure 2B) PPARα expression in HET-Bet liver relative to untreated HET, yet the increase did not reach the WT levels. The upregulated lipogenesis in steatotic liver leads to increased synthesis of TAG, regulated by Dgat1, and Dgat2 (15). Bet treatment modestly reduced the elevated Dgat1 by 20% (*p* = 0.007) and did not affect the elevated Dgat2 (Figure 3C). Taken together, the biochemical, and expression analysis established that supplementation of Bet had a significant lipid-lowering effect on Pcyt2 deficient obesity model.

### Metabolomic comparisons of bet and cho treatments

The ^1^H-NMR spectra were obtained for four types of plasma samples: WT, HET, and HET supplemented with Bet or Cho (Figure 3). This allowed us to compare metabolomic differences between obese and non-obese mouse models (HET vs. WT) and treatments (Bet vs. Cho). When initially compared (representative spectra are shown in Figures 3A,B), there were no major differences in the sample quality and differences among samples were mainly in the peak intensity. A total of 27 specific metabolites were identified within the 0.2 to 8 ppm range and well-defined signature peaks with ppm values indicated in Figure 3C were used for quantification and marker analysis.

### Multivariate data analysis based on global profiling

**Figure 4:**
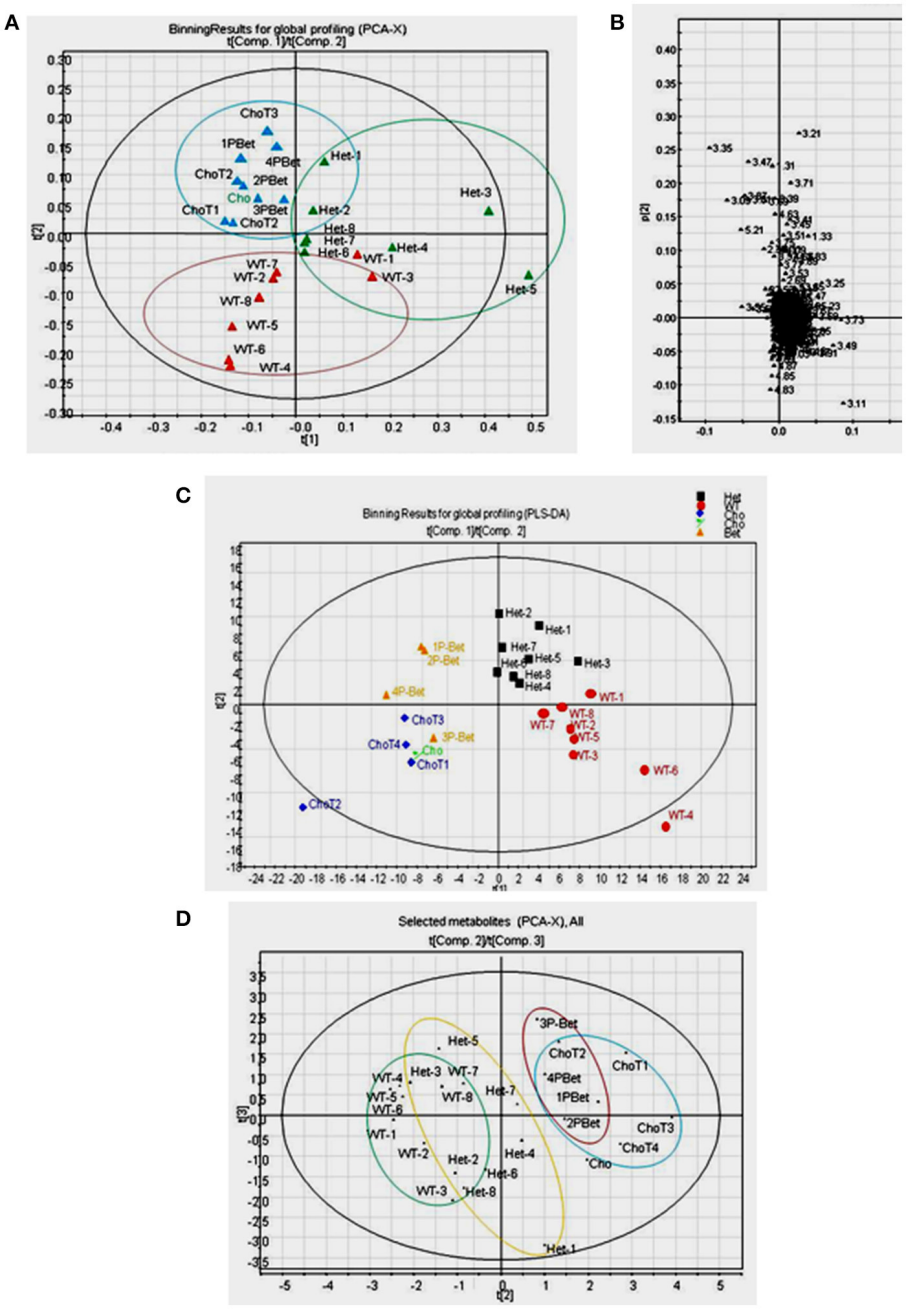
Pattern recognition analyses of ^1^H-NMR spectra for controls and supplemented groups. **(A)** The PCA score plot of ^1^H-NMR spectra for all plasma samples showing a general pattern of separation for Het (*n* = 8), WT (*n* = 8), Cho (*n* = 5)/Bet (*n* = 4) groups; **(B)** The PCA loading plot indicating that only a few bins contributed to the weak separation; **(C)** The PLS-DA score plot of ^1^H-NMR spectra indicating a fairly good general separation for Het, WT, Cho and Bet groups (R^2^X = 0.39, R^2^Y = 0.74, and Q^2^ = 0.12 for 4 components) **(D)** The PCA score plot of selected metabolites showing a general pattern of separation for Het (*n* = 8), WT (*n* = 8), Cho (*n* = 5), and Bet (*n* = 4) groups.

**Figure 5:**
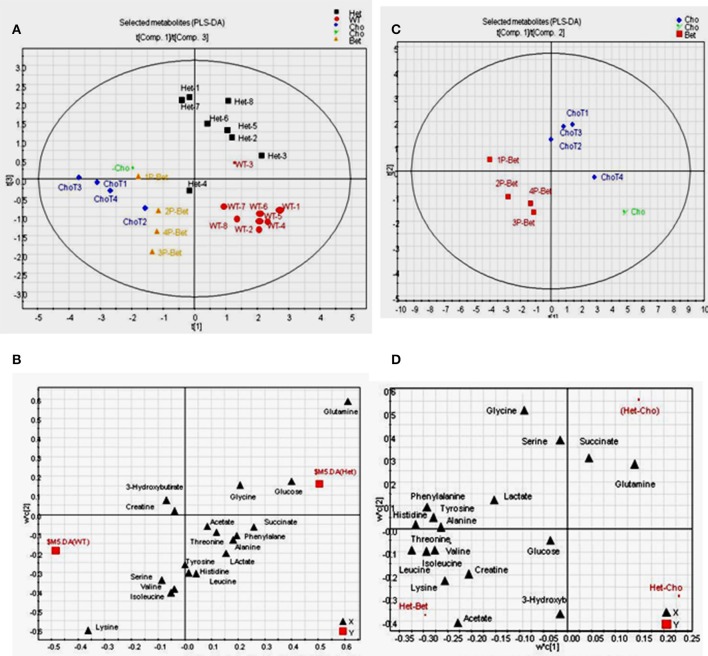
PLS-DA on data from ^1^H-NMR targeted profiling of metabolites for supplemented and control groups **(A)** The score plot on data from the ^1^H-NMR targeted profiling indicating a fairly good separation for Het, WT, Cho and Bet groups (R^2^X = 0.73, R^2^Y = 0.59, and Q^2^ = 0.14 for 4 components); **(B)** The loading plot shows the separation of targeted metabolites, with glutamine, glucose and glycine identified as higher in HET samples and lysine, serine, valine, and isoleucine as higher in WT samples; **(C)** Score plot for Bet and Cho samples showing a good separation for the two treatments (R^2^X = 0.88, R^2^Y = 0.92, and Q^2^ = 0.44 for 4 components); **(D)** The loading plot for Cho and Bet samples identified metabolites that mainly contributed to the separation-acetate, threonine, lysine, creatine, and BCAA (isoleucine, leucine, valine) were higher in Bet groups while glutamine, succinate and serine were higher in Cho groups.

The final ^1^H-NMR data were analyzed using the Principal Component Analysis (PCA) and the Partial Least Squares–Discriminant Analysis (PLS-DA) (Figures 4, 5). The Het (*n* = 8) and WT (*n* = 8) groups were control samples each combined from separate Cho (*n* = 4) and Bet (*n* = 4) trials, and Cho (*n* = 5) and Bet (*n* = 4) groups were treatment dependent sample types.

The spectra were binned and the integral value of each bin was normalized to the standard peak values. The PCA uncovers the general clustering trends and possible outliers in the datasets, without knowing sample groups or class association. With all samples included, the score plot of PCA showed a general pattern of separation for Het (*n* = 8), WT (*n* = 8), Cho (*n* = 5)/Bet (*n* = 4) groups (Figure 4A). WT plasma samples were more similar whereas Het samples varied more. A pattern of separation for Bet and Cho samples was implying distinct treatment effects. The PCA loadings plot revealed that only a few bins contributed to the weak separation (Figure 4B).

By applying class information, we were able to model a better data separation using PLS-DA (Figure 4C). The scores plot of PLS-DA on all samples provided a fairly good separation for Het, WT, Cho and Bet groups (R^2^X = 0.39, R^2^Y = 0.74, and Q^2^ = 0.12 for 4 components). If we only included Het and WT samples in the model, a distinct separation was observed (R^2^X = 0.50, R^2^Y = 0.99, and Q^2^ = 0.75 for 4 components). We were also able to see a cluster separation for Cho and Bet groups (R^2^X = 0.62, R^2^Y = 0.98, and Q^2^ = 0.54 for 4 components). Therefore, the spectral binning analyses provided a general understanding of the separation patterns for 4 types of plasma samples not knowing the sample type.

### Multivariate data analysis based on targeted profiling

Due to the complicated and overlapping nature of the ^1^H-NMR spectra, it is challenging to assign specific metabolites based on the bins that showed to be significant contributors for the cluster separation. Therefore, we used a targeted profiling approach to identify the most relevant metabolites by spectra fitting (Chenomx NMR Suite-Version 7.5 with 304 built-in standards). This enabled us to obtain the plasma concentrations for 27 metabolites for Bet (Table 1) and Cho (Table 2). Among those metabolites, 19 metabolites with relatively higher abundance were selected for multivariate data analysis and comparisons of model types and Cho and Bet treatments (Figures 4D, 5). Similarly to the spectral binning results (Figures 4A–C), and considering the fact that the results are from two separate trials, the initial Cho trial (13), and the Bet trial, 4 groups were fairly well separated in the PCA model (Figure 4D). The Het and WT patterns of separation indicated some overlapping and Cho and Bet groups were fairly separated, suggesting that treatments had distinct effects on Het obesity.

**Table 1:** ^1^H-NMR targeted metabolomics data for betaine treatment.

**Metabolite^*a*^**	**WT**	**HET**	**HET+Bet**	***p*-value**
Ketoglutarate	0.041 ± 0.0096	0.046 ± 0.004	**0.0667** ± 0.008^*#^	0.0291
Hydroxybutyrate	0.229 ± 0.03	0.2098 ± 0.03	**0.1174** ± 0.02^*#^	0.0358
Acetate	0.082 ± 0.01	0.089 ± 0.011	**0.0400** ± 0.009^*#^	0.0196
Methylmalonate	0.031 ± 0.002	0.037 ± 0.006	0.0419 ± 0.008	0.6371
Betaine	0.048 ± 0.004	0.053 ± 0.005	0.056 ± 0.0085	0.7624
Choline	0.0175 ± 0.0007	0.0171 ± 0.002	0.01985 ± 0.003	0.4488
Creatine	0.143 ± 0.008	0.141 ± 0.008	0.1260 ± 0.01	0.2886
DMG	0.009 ± 0.0009	**0.0064** ± **0.0005** ^*^	**0.039** ± 0.0002^*#^	< 0.0001; < 0.0001
Sarcosine	0.0096 ± 0.002	0.0088 ± 0.0008	**0.0030** ± 0.00049^*#^	0.0007
Taurine	0.5166 ± 0.038	0.6144 ± 0.05	**0.3287** ± 0.14^*#^	0.0120
Glucose	10.08 ± 0.68	**12.96** ± 1.019^*^	**12.60** ± **0.95** ^*^	0.0472, 0.0439
Lactate	6.402 ± 0.569	7.299 ± 1.04	8.472 ± 1.66	0.5454
Glutamine	0.4742 ± 0.0178	**0.6143** ± **0.019** ^*^	**0.5338** ± **0.036**^#^	0.0273, 0.0401
Glycerol	1.185 ± 0.214	**0.7197** ± **0.06** ^*^	**2.151** ± **0.458** ^*#^	0.0125, 0.0032
Succinate	0.1614 ± 0.02	0.2568 ± 0.07	**0.3464** ± 0.12^*^	0.0372
Glycine	0.2540 ± 0.01	0.2764 ± 0.017	0.2899 ± 0.02	0.6626
Leucine	0.1422 ± 0.015	0.1466 ± 0.019	0.1694 ± 0.02	0.5022
Isoleucine	0.1077 ± 0.01	0.1013 ± 0.015	0.1141 ± 0.01	0.6104
Histidine	0.062 ± 0.003	0.062 ± 0.005	**0.08** ± **0.005** ^*#^	0.0460
Lysine	0.289 ± 0.015	**0.2410** ± 0.016^*^	**0.241** ± **0.02** ^*^	0.0289, 0.0301
Serine	0.1777 ± 0.01	0.1649 ± 0.02	0.1733 ± 0.016	0.8111
Phenylalanine	0.0729 ± 0.009	0.08283 ± 0.007	0.0862 ± 0.006	0.7567
Threonine	0.1727 ± 0.01	0.1850 ± 0.018	0.1892 ± 0.013	0.8845
Tyrosine	0.1007 ± 0.01	0.1000 ± 0.01	0.1179 ± 0.008	0.3036
Alanine	0.4561 ± 0.037	0.5208 ± 0.06	**0.6022** ± **0.07** ^*^	0.0319
Valine	0.2425 ± 0.03	0.2320 ± 0.03	0.2582 ± 0.02	0.5796

a mM ± S.E.M; Shown are p-values for one-way ANOVA. Significant differences are in bold and compared relative to WT(^*^)and HET (^#^)groups.

**Table 2:** ^1^H-NMR targeted metabolomics data for choline treatment (13).

**Metabolite ^a^**	**WT**	**HET**	**HET+Cho**	***p*-value**
Ketoglutarate	0.041 ± 0.01	0.046 ± 0.004	0.039 ± 0.01	0.3968
Hydroxybutyrate	0.229 ± 0.035	0.210 ± 0.03	**0.106** ± 0.02^*#^	0.0377
Acetate	0.082 ± 0.015	0.089 ± 0.01	**0.024** ± 0.002^*#^	0.0110
Methylmalonate	0.031 ± 0.003	0.037 ± 0.005	0.031 ± 0.004	0.4810
Betaine	0.048 ± 0.004	0.053 ± 0.005	**0.036** ± **0.004**^#^	0.0397
Choline	0.018 ± 0.001	0.017 ± 0.002	**0.015** ± 0.0004^*^	0.0430
Creatine	0.143 ± 0.01	0.141 ± 0.01	**0.102** ± 0.01^*#^	0.0083
DMG	0.009 ± 0.001	**0.006** ± 0.0005^*^	**0.010** ± 0.0003^*#^	0.0328,0.0482
Sarcosine	0.010 ± 0.002	0.009 ± 0.001	**0.003** ± 0.0004^*#^	0.0002
Taurine	0.517 ± 0.04	0.614 ± 0.05	**0.219** ± 0.06^*#^	0.0105
Glucose	10.08 ± 0.68	**12.96** ± 1.02^*^	**12.24** ± 0.90^*^	0.0467,0.0493
Lactate	6.40 ± 0.57	7.30 ± 1.04	6.99 ± 0.53	0.8313
Glutamine	0.474 ± 0.02	**0.614** ± 0.02^*^	**0.575** ± 0.03^*^	0.0217,0.0326
Glycerol	1.19 ± 0.21	0.72 ± 0.06	**2.26** ± 0.21^*#^	< 0.0001
Succinate	0.161 ± 0.02	0.257 ± 0.07	**0.403** ± 0.06^*^	0.0181
Glycine	0.254 ± 0.01	0.276 ± 0.02	**0.282** ± 0.05^*^	0.0455
Leucine	0.142 ± 0.015	0.147 ± 0.02	0.110 ± 0.005	0.1779
Isoleucine	0.108 ± 0.01	0.101 ± 0.015	0.075 ± 0.005	0.2290
Histidine	0.062 ± 0.003	0.063 ± 0.005	0.061 ± 0.004	0.7811
Lysine	0.289 ± 0.015	**0.241** ± 0.02^*^	**0.190** ± 0.01^*#^	0.0380,0.0248
Serine	0.178 ± 0.015	0.165 ± 0.02	0.180 ± 0.02	0.6545
Phenylalanine	0.073 ± 0.009	0.083 ± 0.007	0.063 ± 0.006	0.3728
Threonine	0.173 ± 0.01	0.185 ± 0.02	**0.124** ± 0.01^*^	0.0423
Tyrosine	0.101 ± 0.01	0.100 ± 0.01	0.080 ± 0.015	0.2768
Alanine	0.456 ± 0.04	0.521 ± 0.06	0.444 ± 0.04	0.3881
Valine	0.242 ± 0.03	0.232 ± 0.03	0.258 ± 0.02	0.3118

a mM ± S.E.M; Shown are P-values for one-way ANOVA. Significant differences are in bold and compared relative to(^*^)WT and(^#^)HET groups.

To investigate metabolites associated with the group separation, we used PLS-DA targeting model as well. With all samples included, a relatively clear cluster separation (R^2^X = 0.73, R^2^Y = 0.59, and Q^2^ = 0.14 for 4 components) was obtained for Het and WT groups (Figure 5A). The loading plot (Figure 5B) established that the main contributing metabolites for the cluster separation were glutamine, glucose, and glycine, which were higher in Het plasma and lysine, serine, valine, and isoleucine, which were higher in the WT plasma. When comparing Bet and Cho samples (Figure 5C), the PLS-DA model also displayed a good cluster separation between those two groups (R^2^X = 0.88, R^2^Y = 0.92, and Q^2^ = 0.44 for 4 components, not shown). The loadings plot established that the key metabolites that contributed to the separation were acetate, threonine, lysine, creatine, branched chain amino acids (BCAA) isoleucine, leucine, and valine, which were higher in Bet group, and glutamine, succinate, and serine, that were higher in Cho group (Figure 5D).

### Individual metabolite analysis from bet trial

The final ^1^H-NMR results from the Bet trial are in Table 1. Each metabolite concentration was compared between non obese (WT), obese (HET), and HET-Bet groups. The Bet treated WT group (WT-Bet) was not analyzed by ^1^H-NMR since Bet did not show significant changes in weight, lipids or gene expression in WT mice (Figures 1, 2). As indicated in Table 1, relative to WT, HET mice have elevated glucose (*p* = 0.04), and glutamine (*p* = 0.03) whereas dimethylglycine (DMG) (*p* < 0.0001), and lysine (*p* = 0.03) were reduced. Bet did not significantly change glucose and lysine levels but reduced glutamine (*p* = 0.04) levels. As could be expected, DMG which is the primary product of Bet demethylation was very abundant in Bet plasma (*p* < 0.0001). Further demethylation to sarcosine was reduced, and sarcosine levels in the Bet group were below WT and HET levels (*p* = 0.0007). The specific Bet effects on the TCA cycle included significant increase in α-ketoglutarate (α-KG) (*p* = 0.03) and reductions in ketone bodies (β-hydroxybutyrate - βHB) (*p* = 0.04) and acetate (*p* = 0.02). The most prominent effect of Bet treatment was the increase in plasma glycerol (*p* = 0.003), a product of adipocyte TAG degradation by lipolysis. Although the majority of plasma amino acids were not modified, Bet increased alanine (*p* = 0.03) and histidine (*p* = 0.05) levels and reduced plasma taurine (*p* = 0.01).

### Comparative metabolite analysis from bet and cho trials

**Figure 6:**
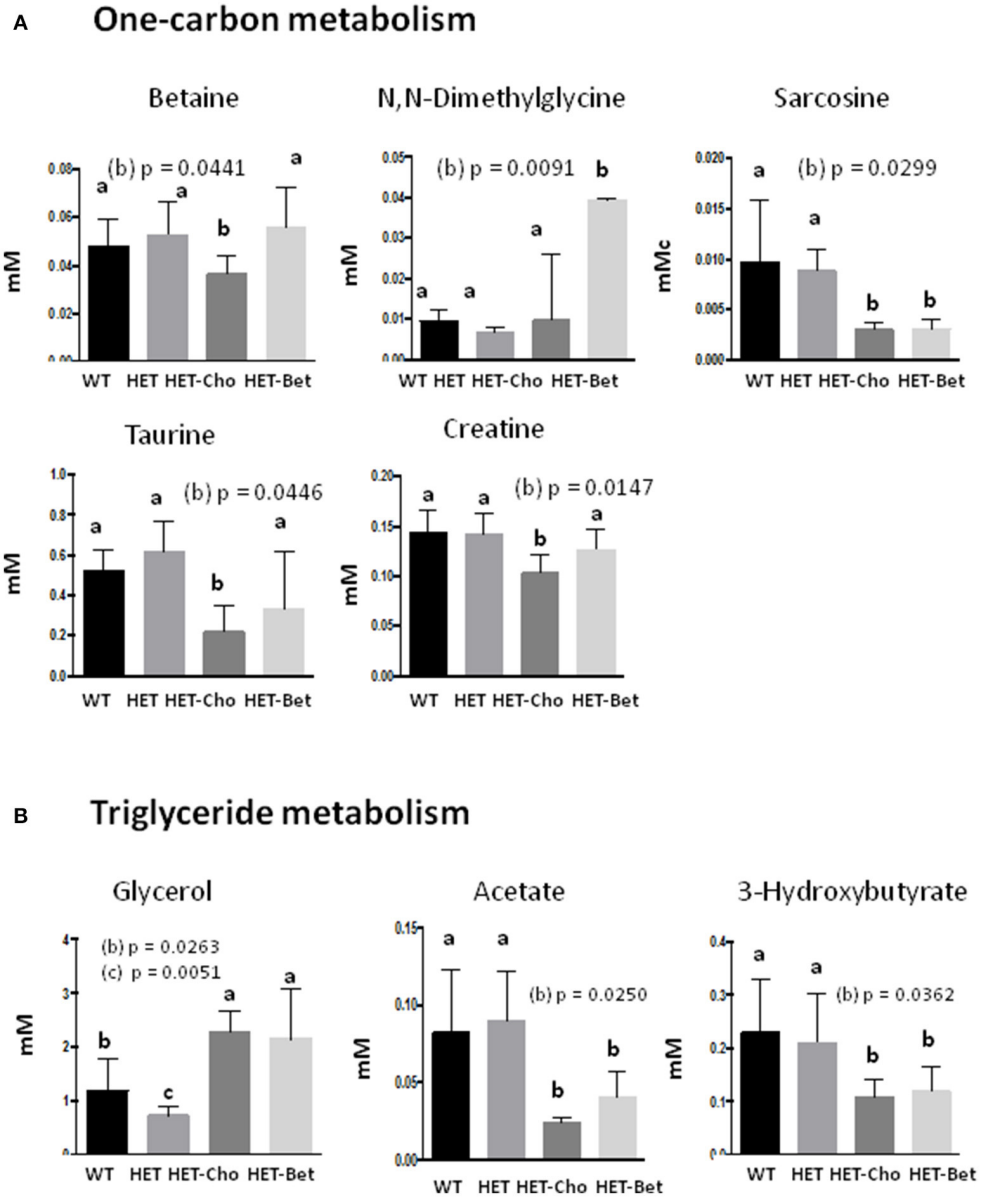
Targeted analysis of the one-carbon cycle and triglyceride metabolism. Discriminant metabolites (mM) modified with genotypes (WT, HET) and treatments (Cho and Bet) included **(A)** the one-carbon metabolism compounds betaine, dimethylglycine, sarcosine, taurine, and creatine; **(B**) the triglyceride metabolism compounds glycerol, acetate and hydroxybutyrate; Data analysis was performed by one-way and two-way ANOVA. The groups are identified by two-way ANOVA; the groups sharing the same letter (*a*) are not statistically different from each other and the groups with different letters (*b* or *c*) are statistically different from other groups at indicated by their *p*-values.

**Figure 7:**
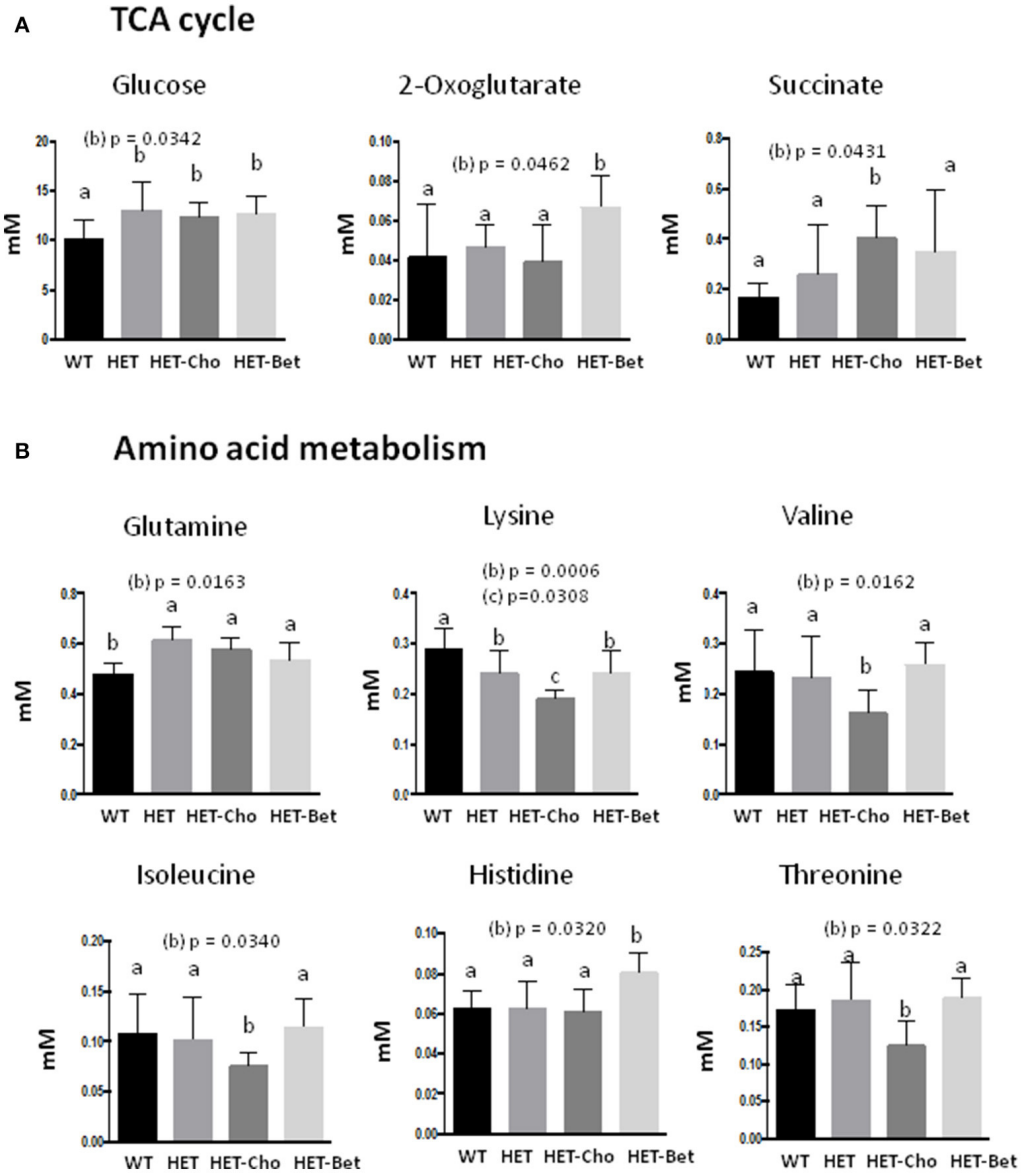
Targeted analyses of the citric acid cycle and amino acid metabolism. Discriminant metabolites (mM) modified with genotypes (WT, HET) and treatments (Cho and Bet) included **(A)** the citric acid cycle compounds glucose, oxoglutarate and succinate; and **(B)** the amino acids glutamine, lysine, valine, isoleucine, histidine, and threonine. Data analysis was performed by two-way ANOVA. The groups sharing the same letter (*a*) are not statistically different from each other and the groups with different letters (*b* or *c*) are statistically different from other groups at indicated by their *p*-values.

Since Bet is the principal product of Cho oxidation, the specific metabolites that could contribute to similarities/differences between Bet (Table 1) and Cho (Table 2) trials were additionally analyzed for untreated (WT and HET) and treated (Bet and Cho) groups by two-way ANOVA (Figures 6, 7). As shown earlier by PLS-DA targeting, the most affected were metabolites from the one-carbon cycle, FA metabolism, amino acid metabolism and TCA cycle. The major similarities between the Cho and Bet trials are that both treatments reduced the generation of sarcosine (*p* = 0.03) from the one-carbon cycle. Cho treatment additionally reduced the levels of Bet (*p* = 0.04), taurine (*p* = 0.04), and creatine (*p* = 0.01). As could be expected, DMG was more abundant (*p* = 0.09) in the Bet trial (Figure 6A). Cho and Bet also similarly affected TAG metabolism, as indicated by 2-fold elevation in plasma glycerol relative to the WT plasma (*p* = 0.026). The similar effect of Cho and Bet on TAG metabolism was also demonstrated by ~2-fold reduction in acetate (*p* = 0.025) and β-HB (*p* = 0.036) in both treatments (Figure 6B).

The specific effect of Cho treatment on the TCA cycle was the accumulation of succinate (*p* = 0.04), while α-KG specifically accumulated in Bet treated mice (*p* = 0.046) (Figure 7A). Finally, the amino acid metabolic profiles were most differently modified with Cho and Bet. Cho significantly reduced the levels of lysine (*p* = 0.03), isoleucine (*p* = 0.03), valine (*p* = 0.016), and threonine (*p* = 0.03). On the other hand, Bet increased the levels of histidine (*p* = 0.03) and had no significant effect on the remaining amino acids (Figure 7B).

## Discussion

The use of Cho and Bet supplementation for treating obesity and maintaining normal systemic metabolism has yet to be properly demonstrated in human clinical trials. We established that Pcyt2 deficient/HET mice lose body weight after 2 months of Bet supplementation that was attributed to reductions in organ (liver and adipose) lipids and elimination of hypertriglyceridemia. The lipid-lowering effect is a collective result of reduced lipid formation by lipogenesis and stimulated lipid degradation by lipolysis.

In addition to impaired lipid metabolism, numerous other factors also contribute to obesity and hypertriglyceridemia observed in HET mice. These include impaired liver VLDL secretion during fasting as well as facilitated intestinal absorption and chylomicron secretion during feeding in combination with globally impaired TAG degradation by lipolysis (16). Due to the complex nature of obesity and commonalities between the anti-obesity effects of Cho (13) and Bet, we chose to perform ^1^H-NMR-based plasma analysis to compare the systemic effects of Bet and Cho treatments.

**Figure 8:**
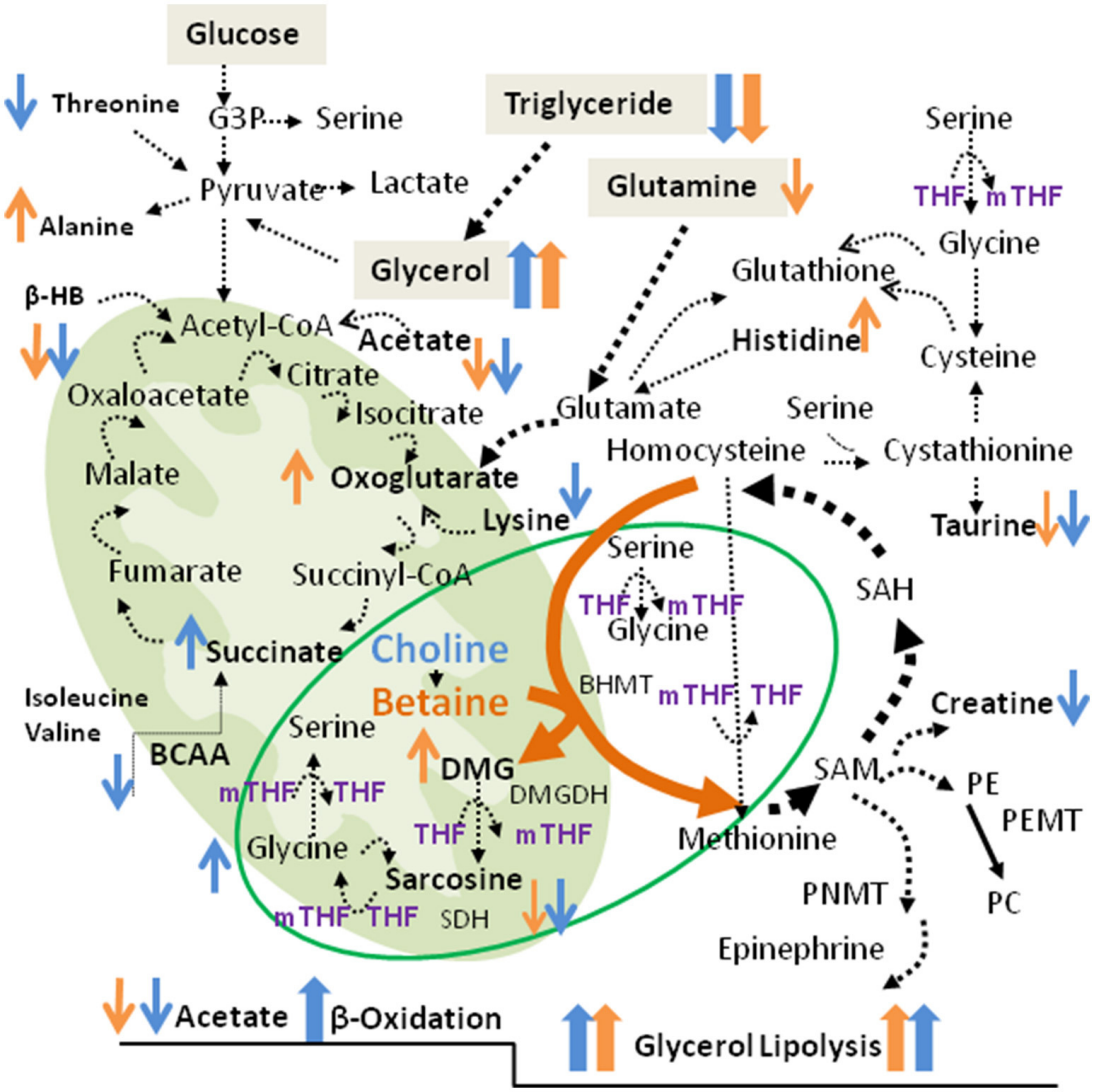
Metabolic pathways associated with betaine and choline supplementation. Shown are the TCA cycle and the Cho/Bet oxidative demethylation pathways in the mitochondria. The cytosolic pathways include the one-carbon metabolism, glycolysis and the amino acid metabolism. The increased and decreased metabolites after Bet and Cho supplementation are indicated in orange and blue, respectively. The metabolic markers specifically modified by Bet and Cho supplementation are indicated in bold.

As displayed in Figure 8, Cho is oxidized to Bet by choline oxidase which requires oxygen and flavin adenine dinucleotide (FAD/FADH_2_) as a cofactor. BHMT then facilitates the donation of one methyl group from Bet to homocysteine, yielding DMG, and methionine respectively. When metabolized to sarcosine (N-methyl glycine), DMG loses one methyl group and sarcosine releases the final methyl group to form glycine (Figure 8). Through DMG and sarcosine oxidative metabolism, Bet contributes methyl groups for folate methylation to generate 5-methyltetrahydrofolate (5mTHF) from tetrahydrofolate (THF) (11). Serine hydroxymethyltransferase (SHMT) can reintroduce the methyl group from 5mTHF to glycine to form serine, and since this is a reversible reaction, serine can donate the methyl group back to folate to generate 5mTHF and regenerate glycine. Therefore, Bet contributes 3 methyl groups via the BHMT reaction and by two sequential oxidative degradation reactions, catalyzed by DMG dehydrogenase (DMGDH) and sarcosine dehydrogenase (SDH). These enzymes are localized in the mitochondrial matrix and require reducing equivalents from FAD/FADH_2_ and folate for function.

Diabetic patients are often deficient in Bet and lose significant amounts of Bet via urination (17). Large scale clinical trials have established that metformin and lifestyle interventions improve plasma Bet and serine status in obese and individuals at high risk for developing diabetes (18). Furthermore, there is a causal relationship between DMG deficiency and diabetes, whereby supplementation of Bet and DMG has demonstrated the potential to be beneficial for the treatment and prevention of diabetes (19).

Somewhat unexpectedly, even after prolonged supplementation neither Cho nor Bet accumulated in circulation, which is an indication of their rapid catabolism and excretion. As expected, Bet treatment increased the circulating levels of DMG and DMG did not accumulate by Cho treatment. The reduction in sarcosine with both treatments was somewhat surprising. The depletion of sarcosine suggests increased oxidative demethylation pathway to generate glycine. Glycine mildly accumulated after Cho treatment (13) but this change was insignificant when compared to Bet treatment. Depletion of taurine by both treatments implies that the trans-sulfuration pathway was more readily utilized for the production of cysteine/glutathione than for the production of taurine. Taurine has long been negatively associated with adiposity, largely due to its ability to promote FA oxidation (20).

^“1^H-NMR results revealed for the first time a direct connection between Cho and Bet oxidative degradation and mitochondrial respiration/TCA cycle (Figure 8). Succinate and α-KG are the most critical metabolites in the TCA cycle as they produce different reducing equivalents required for the respiratory (electron transfer) chain and ATP production. Succinate oxidation by succinate dehydrogenase (SDH, Complex II) derives electrons and produces FADH_2_, while α-KG degradation to succinyl-CoA by α-ketoglutarate dehydrogenase (KGDH) produces a proton, CO2, and NADH. The Cho oxidation and Bet demethylation require different reducing equivalents from the TCA cycle. Choline oxidation to betaine aldehyde by mitochondrial choline dehydrogenase produces FADH2 and derives electrons in a reaction similar to succinate dehydrogenase (SDH, Complex II), when one molecule of choline oxidized to betaine yields 2 molecules of ATP (21). Betaine is also a positive regulator of mitochondrial respiration (22). However, betaine demethylation requires NADH for the formation of THF which then accepts methyl groups from DMG and sarcosine. Those differences in reducing requirements for Cho and Bet degradation (FAD vs. NADH) could explain why their supplementation affected different steps in the TCA cycle.

In addition, the increase in succinate after Cho supplementation could originate from a stimulated amino acid catabolism. BCAA isoleucine and valine, levels were specifically reduced by Cho and not affected by Bet. Threonine and lysine were additional amino acids specifically reduced by Cho treatment (Figure 8). Contrary to Cho, Bet did not cause deficiencies in amino acids, but increased histidine and alanine levels. Bet probably stimulated α-KG formation from glutamate/glutamine since the elevated HET glutamine was significantly reduced by Bet. The α-KG formation from glutamate (glutamine) is catalyzed by KGDH, a key regulatory step in the TCA cycle that determines the rate of electron transport and ATP synthesis (23). Similarly to KGDH, hepatic glutamate dehydrogenase (GLDH) could also increase α-KG, NAD(P)H and consequently ATP (24).”

Alanine (a glucogenic, nonessential amino acid) is the main amino acid produced in skeletal muscle by the glutamate-pyruvate transaminase reaction. Alanine goes to the liver where it can become converted back to pyruvate and then used for glucose production by gluconeogenesis. This inter-organ cycle from muscle glycolysis to liver gluconeogenesis is known as the alanine cycle. The hepatic alanine can also be used for urea synthesis, which also requires the involvement of GLDH (25). Plasma glucose and glutamine levels were not modified and remained high with both Cho and Bet treatments so the elevated alanine specifically seen with Bet but not by Cho treatments probably is not a result of reduced gluconeogenesis. Since Bet specifically increased the circulating levels of alanine, it is more likely that the increased alanine is an indicator of increased muscle glycolysis resulting from Bet treatment.

The HET mice have mildly elevated plasma glucose and impaired muscle insulin signaling but they are not diabetic (12, 14) and Cho supplementation improves HET muscle insulin signaling (14). Paradoxically, the ^1^H-NMR data show that plasma glucose levels remained unmodified with Cho and Bet treatments. The reason for that is because HET mice have elevated glutamine able to maintain glucose levels by gluconeogenic processes. We do not know why glutamine is upregulated in HET mice. As a nitrogen and carbon source, glutamine is involved in regulating the TCA cycle, redox balance and is also a critical nutrient during aerobic glycolysis (26). The “glutamine addiction” of HET mice is probably compensatory to impaired glucose metabolism but could also involve numerous non metabolic processes (27).

The most valuable contribution of Cho and Bet supplementation is their positive effect on reducing lipid mass by stimulating tissue lipolysis. This was evident from an accumulation of the lipolytic product glycerol and reduced acetate levels in the circulation. Glycerol is generally conceived as a measure of adipose tissue lipolysis. The excess glycerol could contribute glucose synthesis via gluconeogenesis but can also participate in energy production via glycerol 3-phosphate (G3P) oxidation. G3P is oxidized in the “G3P shuttle” by G3P dehydrogenases (GPDH) and contributes to the electron transport from cytosolic NADH to mitochondrial respiratory complexes (28).

β-HB is oxidized and converted to acetoacetyl-CoA which is then metabolized via the TCA cycle (Figure 8). β-HB is acutely elevated in diabetic ketoacidosis, liver ischemia and similar disorders characterized by reduced mitochondrial redox function (29). Since HET mice had normal β-HB, Bet and Cho supplementation apparently promoted the use of β-HB as fuel (ketolysis) in extrahepatic tissues like the brain. In addition, changes in circulating β-HB and glycerol were similar between Cho and Bet treatments.

We showed previously that free FAs are not elevated in HET plasma (12). However, ^14^C-oleate radiolabeling of hepatocytes showed elevated FA uptake and reduced liver FA oxidation (12). Injected ^14^C-oleate increasingly incorporated into HET liver TAG and Cho supplementation reduced the oleate incorporation that was followed with reduction in steatosis and obesity (13). The elevated plasma acylcarnitines are direct measure of impaired mitochondrial FA oxidation. In the Cho supplementation study, we provided evidence that Cho reduced the elevated plasma acylcarnitines and therefore improved FA oxidation in HET tissues (13). Although we did not measure plasma acylcarnitines after Bet treatments, we showed that Bet share multiple phenotypic and metabolic similarities with Cho treatments. Both, Bet and Cho reduced hepatic steatosis and adiposity and shared plasma metabolite profiles, including reduced plasma and tissue TAG and increased levels of circulating glycerol altogether indicating the lipid-lowering effect and improved FA utilization in both treatments.

Taken together, we suggest that the main anti-obesity effect of Cho and Bet supplementation was stimulation of TAG degradation by lipolysis, which impacts metabolism in a manner that mimics energy deficient states such as fasting. An important question remains: how is the breakdown of TAG mediated in extrahepatic tissues such as adipose tissue when the oxidative metabolism of Cho and Bet mainly occurs in liver mitochondria? One possibility is that by correcting the abnormalities of the liver steatosis, Cho and Bet improved general metabolic and hormonal status of HET mice (30). This is demonstrated by the lipid gene expression profile, the enhanced insulin signaling and AMPK activity in the adipose tissue (13) and skeletal muscle of Cho treated HET mice (14). Cho and Bet exhibited similar reducing effects on the key lipogenic transcription factor Srebp1 known to be involved in adipocyte differentiation promoting an anti-inflammatory and insulin sensitive adipocyte phenotype (13, 14). Fibroblast growth factor 21 (Fgf21) is a cytokine released from the liver which is a potent regulator of adipocyte function and lipolysis. Fgf21 stimulates lipolysis by a systemic release of catecholamines (31) and is critical for attenuation of obesity (32). It was shown previously that Bet supplementation stimulates hepatic and circulating Fgf21 levels and improves FA oxidation and energy balance in rodent models of diet induced obesity (33, 34). Here, we have evidence to suggest how Cho improves metabolic health by the mitochondrial Bet degradation pathway. The effect of Cho supplementation on Fgf21 levels is not known but it will be important to be established in the future.

In conclusion, our study demonstrates that Cho and Bet administration is beneficial for obese and insulin resistant HET mice. The protection from lipid accumulation associated with reductions in liver steatosis and increased lipid breakdown in adipocytes is attributed to increased demands for degradation and elimination of supplemented Cho and Bet. The data provide new insight on how Cho and Bet supplementations could be beneficial for the treatment of obesity and type 2 diabetes due to their valuable metabolic effects on lipolysis, TCA cycle and mitochondrial oxidative phosphorylation.

## Methods

### Betaine treatments

CTP: phosphoethanolamine cytidylyltransferase (Pcyt2) littermate controls (WT) and heterozygous (Pcyt2^+/−^, HET) mice were generated as previously described (15). Ten month old male mice (*n* = 8 in each group) were exposed to a 12 h light/dark cycle and allowed access to a standardized diet (Harlan Teklad S-2335). The control groups, WT (lean) and HET (obese), had access to water and the treatment groups (WT-Bet and HET-Bet) had access to 1% Bet supplemented water for a total of 8 weeks. Water intake was measured daily and body weight was measured every 7 days. All mice were euthanized after 8 weeks and liver, plasma and adipose tissue collected. All procedures were approved by the University of Guelph's Animal Care Committee and were in accordance to guidelines of the Canadian Council on Animal Care.

### Choline treatments

Ten month old mice were divided into 3 groups (*n* = 6–10 per group): WT (lean), HET (obese), and HET treated with Cho (HET+Cho). HET+Cho mice had access to water containing 2% choline for 4 weeks. Water intake was recorded daily and body weight was recorded weekly. Mice were euthanized after 4 weeks of choline treatment (13).

### Plasma collection and lipid analysis

Blood was collected through cardiac puncture and dispensed into three EDTA coated tubes stored on ice and spun down at 3,000 g for 10 min at 4°C. The obtained plasma (80–100μl) was transferred into sterilized Eppendorf tubes and kept at −80°C. Plasma TAG was assayed using a standard kit (Sigma TR0100 Kit).

### Liver histological analyses

Fresh livers and adipose tissue were dissected and fixed in 10% formalin in PBS and embedded in parafilm. Sections of tissue (10 μm in thickness) were dewaxed in xylene and rehydrated by a series of ethanol washes and stained with hematoxylin and eosin. The analysis was performed with a computerized image analysis system comprised of a photomicroscope and digital camera (Olympus Biological Microscopes: FSX100; Carl Zeiss Microscopy, Oberkochen, Germany) and software (FSX-100; Olympus global).

### Total RNA extraction and RT-PCR

A total of 100 mg of tissue was homogenized in TRIzol reagent (Invitrogen) and mRNA extracted according to the manufacturer's protocol. cDNA was synthesized from 4 μg of mRNA using a poly-dT primer and Superscript II reverse transcriptase (Invitrogen). The genes involved in TAG synthesis and degradation were analyzed. Diacylglycerol acyltransferase 1 and 2 (Dgat1/2), sterol regulatory element binding protein 1c (Srebp1c), FA synthase (Fas), peroxisome proliferator activated receptor (Pparα), and stearoyl-CoA desaturase (Scd1) were amplified using gene specific primers and the following PCR parameters: 94°C for 45 s, 57°C for 30 s, and 72°C for 45 s for 32 cycles. Glyceraldehyde-3-phosphate dehydrogenase (Gapdh) control was amplified using the following cycle parameters: 94°C for 45 s, 58°C for 30 s, and 72°C for 45 s for 30 cycles. The band density was quantified using Image J densitometry software (NIH).

### Statistical analysis

All data are expressed as a mean ± SEM. The statistical analysis for Bet trial was performed by one-way ANOVA with Dunnett's *post-hoc* test as compared to WT lean control. Two-way ANOVA with Turkey *post-hoc* test was employed to compare the effects of genotype (WT, HET) and treatment (WT-Bet and HET-Bet) on tissue weight, lipid content, and gene expression. Two-way ANOVA with Turkey *post-hoc* test was also employed to compare genotype (WT, HET) and treatment (HET-Bet and HET-Cho) on plasma metabolites using GraphPad Prism 4.

### ^1^H-NMR metabolomic profiling

#### Sample preparation and data acquisition

The plasma samples from untreated HET obese mice (Het-1-8) included 4 samples for Bet trial and 4 samples for Cho trial and the WT lean control (WT-1-8) samples included 4 samples for Bet trial and 4 samples for Cho trial. Additional samples included 4 Bet treated HET (Bet-1-4) and 5 Cho treated HET (Cho-1-5) plasma. The samples were stored at −80°C and right before use thawed in ice water, filtered through prewashed 3 KDa membranes (EMD Millipore, Billerica, MA, USA) and centrifuged at 14,000 g, 4°C for 40 min. For the batch of HET and WT samples, 30 μl plasma was mixed with 30μl 0.5 mM DSS-d6 standard, and 100 mM phosphate buffer (pH 7.0) in D_2_O. For Cho and Bet samples, 30 μl plasma was mixed with 2 μl 10 mM DSS standard and 45μl 100 mM phosphate buffer solution (pH 7.0) in D_2_O. For NMR experiments, 45μl solution of each sample was transferred to a 1.7 mm NMR tube. Chemical shift and concentration standards 4,4-Dimethyl-4-silapentane-1-sulfonic acid (DSS) and DSS-d6 were from Sigma-Aldrich Canada Ltd, Oakville.

NMR spectra were acquired on a Bruker Avance III 600 MHz NMR spectrometer (Bruker Biospin Ltd., Milton, ON, Canada) which has a ^1^H operating of 600.284 MHz. Spectra were obtained with a 1.7 mm inverse gradient probe with automatic tuning and shimming and the temperature was controlled to 25.00 ± 0.02°C on each sample. When a sample was placed in the magnet, it was allowed to remain undisturbed for 300 s to obtain thermal equilibrium. After this time, a simple probe image was acquired to ensure the each sample was in the probe correctly before the subsequent experiments. The π/2 was calibrated on each sample (around 7.25μs) using pulsecal (a macro in the Bruker software). A presaturation experiment was performed on each sample consisting of 8 scans, using a presaturation/delay time of 4 s, the calibrated π/2, an acquisition time of 2.72 s (64k points), and a sweep width of 20.02 ppm (12019 Hz), and 8 scans. 1D NOESY spectra with pre-saturation of the water signal during both the relaxation delay and mixing time were acquired with 128 scans, the calibrated π/2, an acquisition time of 2.72 s (64 K points), a sweep width of 20.02 ppm (12019 Hz), a mixing time of 100 ms, and a relaxation delay of 4 s. The ^1^H-NMR chemical shifts in the spectra were referenced to 0.00 ppm with respect to the internal DSS or DSS-d6 standards. All the data was acquired and processed using TopSpin NMR data acquisition and processing Software (Bruker Biospin Ltd., East Milton, ON) to generate proton NMR spectra for comparison.

#### Data processing and statistical analysis

To prepare dataset for multivariate data analysis, the raw ^1^H-NMR data was also processed using Chenomx NMR Suite (Chenomx Inc, Edmonton, AB). For global profiling using spectral binning, the FID file of each sample was imported, with phase and baseline corrected, and chemical shift calibrated by referencing to DSS or DSS-d6 standards. The spectra were then binned into 0.02 ppm buckets from 0–10 ppm, with selected regions excluded for water (4.73–4.82 ppm) and DSS (−0.60–0.66, 1.72–1.80, 2.88–2.94, 4.73–4.82 ppm).

For targeted profiling, the processed spectra were loaded into the Profiler module, and fit against 304 standard compounds built in the software. Using DSS or DSS-d6 concentration in the sample as reference, the concentrations for metabolites profiled with confidence were obtained through the fitting process. All the spectral binning and targeted profiling data, after being adjusted in response to different diluting factors for the two experiment batches (× 2 for Het/WT samples, and × 2.57 for the rest) was exported as MS Excel dataset for further analysis. The datasets from spectral binning and targeted profiling were imported to SIMCA-P+ (Umetrics, Umea, Sweden) for conducting principal components analysis (PCA) and partial least squares discriminant analysis (PLS-DA). The bins or metabolites concentrations were all mean-centered and Pareto-scaled prior to the analysis.

## Data availability

The datasets analyzed during the current study are available from the corresponding author on reasonable request.

## Ethics statement

This study was carried out in accordance with the recommendations of name of guidelines, name of committee. The protocol was approved by the name of committee.

## Author contributions

SS performed and designed the research study. AT and JZ performed data analysis. MB designed the research study, analyzed the data, and wrote the manuscript.

### Conflict of interest statement

The authors declare that the research was conducted in the absence of any commercial or financial relationships that could be construed as a potential conflict of interest.
